# Data Obfuscation Through Latent Space Projection for Privacy-Preserving AI Governance: Case Studies in Medical Diagnosis and Finance Fraud Detection

**DOI:** 10.2196/70100

**Published:** 2025-03-12

**Authors:** Mahesh Vaijainthymala Krishnamoorthy

**Affiliations:** 1 Stelmith, LLC, 2333 Aberdeen Pl, Carollton, TX, 75007, United States, 1 9459001314

**Keywords:** privacy-preserving AI, latent space projection, data obfuscation, AI governance, machine learning privacy, differential privacy, k-anonymity, HIPAA, GDPR, compliance, data utility, privacy-utility trade-off, responsible AI, medical imaging privacy, secure data sharing, artificial intelligence, General Data Protection Regulation, Health Insurance Portability and Accountability Act

## Abstract

**Background:**

The increasing integration of artificial intelligence (AI) systems into critical societal sectors has created an urgent demand for robust privacy-preserving methods. Traditional approaches such as differential privacy and homomorphic encryption often struggle to maintain an effective balance between protecting sensitive information and preserving data utility for AI applications. This challenge has become particularly acute as organizations must comply with evolving AI governance frameworks while maintaining the effectiveness of their AI systems.

**Objective:**

This paper aims to introduce and validate data obfuscation through latent space projection (LSP), a novel privacy-preserving technique designed to enhance AI governance and ensure responsible AI compliance. The primary goal is to develop a method that can effectively protect sensitive data while maintaining essential features necessary for AI model training and inference, thereby addressing the limitations of existing privacy-preserving approaches.

**Methods:**

We developed LSP using a combination of advanced machine learning techniques, specifically leveraging autoencoder architectures and adversarial training. The method projects sensitive data into a lower-dimensional latent space, where it separates sensitive from nonsensitive information. This separation enables precise control over privacy-utility trade-offs. We validated LSP through comprehensive experiments on benchmark datasets and implemented 2 real-world case studies: a health care application focusing on cancer diagnosis and a financial services application analyzing fraud detection.

**Results:**

LSP demonstrated superior performance across multiple evaluation metrics. In image classification tasks, the method achieved 98.7% accuracy while maintaining strong privacy protection, providing 97.3% effectiveness against sensitive attribute inference attacks. This performance significantly exceeded that of traditional anonymization and privacy-preserving methods. The real-world case studies further validated LSP’s effectiveness, showing robust performance in both health care and financial applications. Additionally, LSP demonstrated strong alignment with global AI governance frameworks, including the General Data Protection Regulation, the California Consumer Privacy Act, and the Health Insurance Portability and Accountability Act.

**Conclusions:**

LSP represents a significant advancement in privacy-preserving AI, offering a promising approach to developing AI systems that respect individual privacy while delivering valuable insights. By embedding privacy protection directly within the machine learning pipeline, LSP contributes to key principles of fairness, transparency, and accountability. Future research directions include developing theoretical privacy guarantees, exploring integration with federated learning systems, and enhancing latent space interpretability. These developments position LSP as a crucial tool for advancing ethical AI practices and ensuring responsible technology deployment in privacy-sensitive domains.

## Introduction

### Background

The rapid advancement and widespread adoption of artificial intelligence (AI) across critical sectors of society have ushered in an era of unprecedented data analysis and decision-making capabilities. From health care diagnostics to financial fraud detection, AI systems are processing increasingly large volumes of sensitive personal data. However, this progress has been accompanied by growing concerns about privacy, data protection, and the potential misuse of personal information.

The tension between leveraging data for AI advancements and protecting individual privacy has become a central challenge in the field of AI governance. Traditional approaches to data privacy, such as anonymization and differential privacy, often struggle to balance the trade-off between privacy protection and data utility. As AI systems become more sophisticated, there is an urgent need for novel privacy-preserving techniques that can protect sensitive information without significantly compromising the performance of AI models.

In this research, we introduce data obfuscation through latent space projection (LSP), a novel privacy-preserving technique designed to address these challenges. LSP leverages recent advancements in representation learning and adversarial training to create a privacy-preserving data transformation pipeline. By projecting raw data into a latent space and then reconstructing it with carefully controlled information loss, we aim to obfuscate sensitive attributes while preserving the overall structure and relationships within the data that are crucial for AI model performance.

This research makes several significant contributions to the field of privacy-preserving machine learning. At the core of this work, we develop and present a comprehensive latent space projection framework, providing detailed insights into its theoretical underpinnings, architectural design, and practical implementation considerations. We advance the field’s measurement capabilities by introducing innovative metrics specifically designed to evaluate the critical balance between privacy protection and data utility in latent space representations. Through rigorous experimentation on established benchmark datasets, we demonstrate that LSP consistently outperforms traditional privacy-preserving approaches across multiple performance dimensions.

To bridge the gap between theory and practice, we showcase LSP’s real-world effectiveness through 2 critical case studies in highly sensitive domains: cancer diagnosis and financial fraud detection. Understanding the practical constraints of deployment, we conduct thorough analyses of LSP’s operational characteristics, including latency and computational resource requirements. Finally, we explore the broader implications of our work, examining how LSP contributes to the responsible development of AI systems and aligns with emerging global AI governance frameworks, providing a foundation for future privacy-preserving AI applications.

### The Privacy Challenge in AI

The exponential growth of data and the increasing sophistication of AI models have led to significant advancements in various fields. However, this progress has also raised critical privacy concerns [1]. AI models, particularly deep learning architectures, often require vast amounts of data to achieve high performance. This data frequently contains sensitive personal information, ranging from medical records to financial transactions.

The potential for privacy breaches in AI systems is multifaceted and detailed in the following sections.

### Data Breaches

Large datasets used for AI training are attractive targets for cyberattacks, potentially exposing the sensitive information of millions of individuals[2,3].

### Model Inversion Attacks

Sophisticated attacks can potentially reconstruct training data from model parameters, compromising the privacy of individuals in the training set [4].

### Membership Inference

These attacks aim to determine whether a particular data point was used in training a model, which can reveal sensitive information about individuals [5].

### Attribute Inference

Even when direct identifiers are removed, AI models may inadvertently learn and expose sensitive attributes of individuals in their training data [6].

### Unintended Memorization

Neural networks have been shown to sometimes memorize specific data points from their training set, potentially exposing sensitive information during inference [7].

These privacy risks are not merely theoretical. High-profile incidents of privacy breaches and misuse of personal data have eroded public trust in AI systems and raised regulatory scrutiny. Consequently, there is an urgent need for robust privacy-preserving techniques that can mitigate these risks while allowing AI to deliver its potential benefits to society.

### Existing Privacy-Preserving Techniques

Several approaches have been developed to address privacy concerns in AI.

#### K-Anonymity

Introduced by Sweeney [8], k-anonymity ensures that each record in a dataset is indistinguishable from at least k-1 other records with respect to certain identifying attributes. Although effective for simple datasets, k-anonymity struggles with high-dimensional data common in modern AI applications.

#### Differential Privacy

Developed by Dwork et al [9], differential privacy provides a formal framework for quantifying and limiting the privacy risk of statistical queries on datasets. It has been successfully applied to various machine learning algorithms [10,11] but often introduces a significant trade-off between privacy and model utility.

#### Homomorphic Encryption

This technique allows computations to be performed on encrypted data without decryption [12]. Although providing strong privacy guarantees, homomorphic encryption incurs substantial computational overhead, making it impractical for many real-time AI applications.

#### Federated Learning

Proposed by McMahan et al [13], federated learning allows models to be trained on decentralized data without directly sharing raw information. However, it can still be vulnerable to certain types of privacy attacks and faces challenges in scenarios requiring centralized data analysis.

#### Synthetic Data Generation

Techniques like differentially private generative adversarial networks (GANs) [14] aim to generate synthetic datasets that preserve statistical properties of the original data while providing privacy guarantees. However, these methods often struggle to capture complex relationships present in real-world data.

Although each of these approaches has its merits, they all face limitations when applied to the complex, high-dimensional datasets typical in modern AI applications. Many struggle to provide strong privacy guarantees without significantly degrading model performance or incurring prohibitive computational costs.

### The Promise of Latent Space Approaches

Recent advancements in representation learning, particularly in the field of deep learning, have opened new avenues for privacy-preserving data analysis [15]. Latent space models, such as autoencoders and variational autoencoders [16], have demonstrated a remarkable ability to learn compact, abstract representations of complex data.

### Latency Characteristics

LSP’s latency profile can be broken down into three main components: (1) encoding latency (the time taken to project input data into the latent space), (2) processing latency (the time required to perform operations, eg, machine learning tasks, in the latent space), and (3) decoding latency (the time needed to reconstruct data from the latent space, if required).

### Performance Optimization Characteristics

These latent representations offer several potential advantages for privacy-preserving AI. Several optimizations contribute to LSP’s improved latency and overall performance:

1. Dimensionality reduction: By projecting data into a lower-dimensional latent space, LSP reduces the computational complexity of subsequent operations, so irrelevant or sensitive features can be naturally obscured. This is particularly beneficial for high-dimensional data like images or complex time series.
2. Parallel processing: The encoder and decoder networks in LSP can leverage the parallel processing capabilities of modern GPUs, significantly speeding up the projection and reconstruction processes.
3. Caching mechanisms: For scenarios where the same data are processed multiple times, LSP implementations can cache latent representations, eliminating the need for repeated encoding.
4. Model compression: Techniques such as pruning and quantization can be applied to the LSP networks, reducing their size, and improving inference speed without significantly impacting privacy or utility.
5. Adaptive computation: LSP can be implemented with adaptive computation techniques, where the depth or width of the network is dynamically adjusted based on the complexity of the input, further optimizing performance.
6. Disentanglement: Advanced techniques in representation learning aim to disentangle different factors of variation in the data, potentially allowing for selective obfuscation of sensitive attributes.
7. Nonlinear transformations: The complex, nonlinear mappings learned by deep neural networks can potentially create representations that are difficult to invert without knowledge of the encoding process.
8. Compatibility with deep learning: Latent space approaches integrate naturally with deep learning architectures, allowing for end-to-end privacy-preserving AI pipelines.

Building on these insights, our proposed LSP technique aims to leverage the power of latent space representations to create a robust, flexible framework for privacy-preserving AI. By combining ideas from representation learning, adversarial training, and information theory, LSP seeks to overcome the limitations of existing approaches and provide a more effective solution to the privacy challenges in modern AI systems.

### Related Work

Privacy-preserving techniques in AI have garnered significant attention, particularly as regulations such as the General Data Protection Regulation (GDPR) and California Consumer Privacy Act (CCPA) come into force. Existing methods provide foundational solutions but have limitations when applied to large-scale data systems.

#### Differential Privacy

Differential privacy, introduced by Dwork et al [17], is a method that adds calibrated noise to datasets or model outputs to obscure individual data points while preserving the overall distribution. Despite its utility, differential privacy often introduces trade-offs between privacy and model accuracy, particularly when applied to complex, high-dimensional data [18].

#### Homomorphic Encryption

Homomorphic encryption allows computations to be performed on encrypted data without decrypting it [12]. Although this approach is highly secure, its computational overhead makes it impractical for large-scale machine learning models that require real-time processing or high-volume datasets [19].

#### Federated Learning

Federated learning, proposed by McMahan et al [13], ensures that raw data remains decentralized, with models trained on local devices instead of centralized servers. However, this technique is not immune to privacy risks, as model gradients or weights exchanged between devices can still leak sensitive information [20,21].

#### Generative Models for Privacy

Recent work has explored the use of generative models, such as GANs, for creating synthetic data that preserves privacy [22]. Although promising, these approaches often struggle with mode collapse and may not fully capture the complexity of real-world data distributions.

LSP builds upon these existing approaches while addressing their limitations. By learning privacy-preserving latent representations, LSP aims to provide a more flexible and efficient solution for data obfuscation that can be applied across various domains and AI tasks.

## Methods

### Data Obfuscation Through LSP

In this section, we present the details of our LSP framework for privacy-preserving data obfuscation. We begin by outlining the key principles behind LSP, then describe the network architecture and training procedure.

### Principles of LSP

The core idea behind LSP is to transform raw data into a latent space where sensitive information is obscured, yet essential features for downstream AI tasks are retained. This is achieved through the following key principles.

- Feature preservation: The latent representation should maintain sufficient information for relevant AI tasks, ensuring high utility of the obfuscated data.
- Adversarial privacy: We employ adversarial training to make it difficult for an attacker to recover sensitive information from the latent representation.
- Task-agnostic design: The LSP framework is designed to be adaptable to various data types and downstream tasks without requiring significant modifications.

### Network Architecture

**Figure 1.**
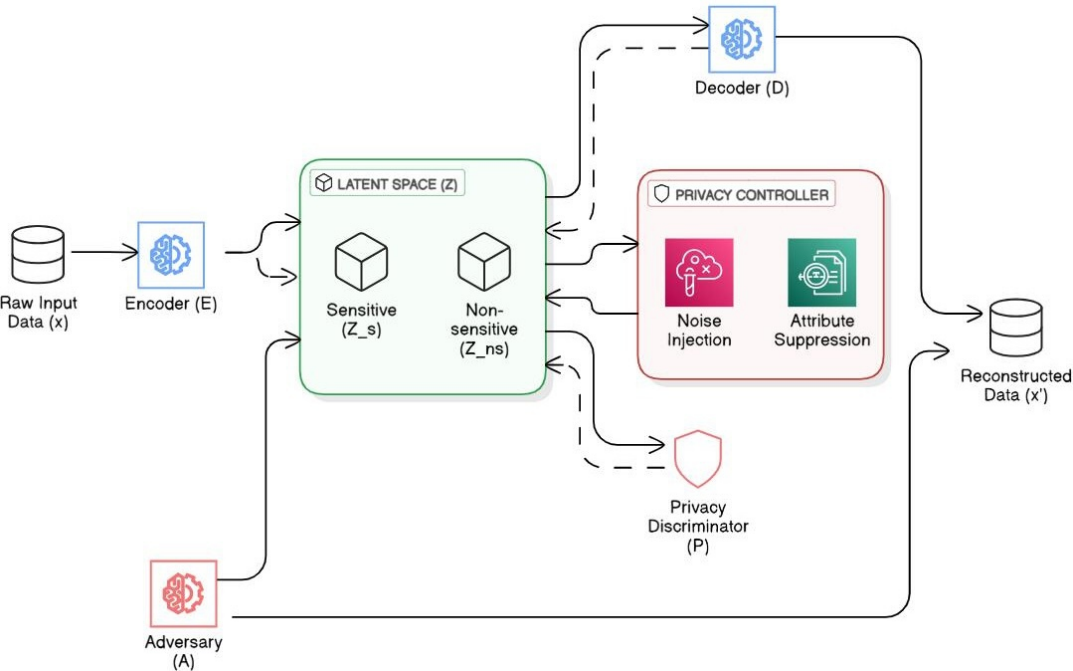
Latent space projection system architecture (network diagram).

Figure 1 depicts the flow of data through the LSP framework. The input data x is first passed through the encoder network E, which projects it into a latent space representation z. This latent representation is then processed by the decoder network D to reconstruct the input, producing x’. Simultaneously, the privacy discriminator P attempts to extract sensitive information s from the latent representation z. The framework is trained adversarial to optimize the trade-off between reconstruction accuracy and privacy protection.

The LSP framework consists of three main components: an encoder network, a decoder network, and a privacy discriminator. These components work together to create privacy-preserving latent representations of the input data. Figure 1 illustrates the overall architecture of the LSP framework.

### Encoder Network

The encoder network E (X → Z) maps the input data x ∈ X to a latent representation z ∈ Z. We implement E as a deep neural network with an architecture tailored to the specific data type.

For image data, the encoder architecture uses a progressive series of convolutional layers with expanding filter sizes, beginning at 32 and scaling up through 64, 128, and 256 filters. Each convolutional operation is augmented by batch normalization and leaky rectified linear unit (ReLU) activation functions to improve training stability and introduce nonlinearity. The network incorporates strided convolutions or max pooling operations strategically placed throughout the architecture to achieve spatial downsampling of the feature maps. The encoding process culminates in fully connected layers that compress the processed features into the final latent representation, effectively capturing the essential characteristics of the input data in a lower-dimensional space.

For text data, the text encoder’s architecture begins with an embedding layer that transforms input tokens into dense vector representations. At its core, the model utilizes a transformer encoder equipped with multihead self-attention layers to capture complex relationships between tokens in the input sequence. The architecture incorporates layer normalization and residual connections between transformer blocks to facilitate stable training and effective gradient flow. The encoding process concludes with a pooling operation, specifically mean pooling, followed by fully connected layers that produce the final encoded representation of the text input.

The latent space Z is structured as Z=Z_s ⊕ Z_ns, where Z_s represents the subspace for sensitive information and Z_ns for nonsensitive information. This separation is enforced through the loss functions and architecture design, which we will discuss in detail in the training procedure section.

### Decoder Network

The decoder network D (Z → X’) reconstructs the input data from the latent representation. Its architecture mirrors that of the encoder.

For image data, the decoder architecture begins with fully connected layers that transform the latent space representation back into a spatial format, setting the foundation for image reconstruction. This is followed by a cascade of transposed convolutional layers with progressively decreasing filter sizes, systematically expanding the spatial dimensions while refining feature details. Each transposed convolutional layer incorporates batch normalization and ReLU activation functions to maintain training stability and introduce necessary nonlinearities. The network uses upsampling operations, utilizing either nearest-neighbor or bilinear interpolation techniques, to gradually restore the spatial resolution of the features. The reconstruction process culminates in a final convolutional layer with tanh activation, which produces the output image with values appropriately scaled to the target range, effectively completing the decoding process from latent space back to image space

For text data, the text decoder’s architecture initiates with fully connected layers that transform the latent space representation into a sequence format suitable for text generation. At its heart, the model uses a transformer decoder equipped with multihead attention layers, enabling the network to effectively capture complex dependencies and relationships within the generated sequence. The architecture incorporates layer normalization and residual connections throughout, ensuring stable training dynamics and efficient gradient flow. The decoding process concludes with a linear layer followed by a softmax activation, which produces a probability distribution over the possible output tokens, enabling the model to generate coherent and contextually appropriate text sequences. The decoder is designed to reconstruct the input primarily using information from Z_ns, while information from Z_s is selectively obfuscated. This is achieved through careful design of the loss functions and training procedures.

### Privacy Discriminator

The privacy discriminator P (Z → S) attempts to recover sensitive information s ∈ S from the latent representation z. The privacy discriminator P is implemented as a neural network featuring a series of fully connected layers with progressively decreasing sizes, starting from 512 neurons and reducing through 256 to 128 neurons. Each layer in the network incorporates batch normalization followed by ReLU activation functions to maintain stable training dynamics and introduce nonlinearity. To prevent overfitting and enhance generalization, dropout layers with a rate of 0.3 are strategically integrated throughout the architecture.

The network culminates in a final layer whose activation function is specifically chosen to match the nature of the sensitive attribute being protected, using sigmoid activation for binary attributes or softmax activation for categorical variables, effectively enabling the network to learn and identify potential privacy leakage in the latent representations. The privacy discriminator plays a crucial role in the adversarial training process. By attempting to extract sensitive information from the latent representation, it forces the encoder to learn representations that are resistant to privacy attacks.

### Information Flow and Gradient Propagation

**Figure 2.**
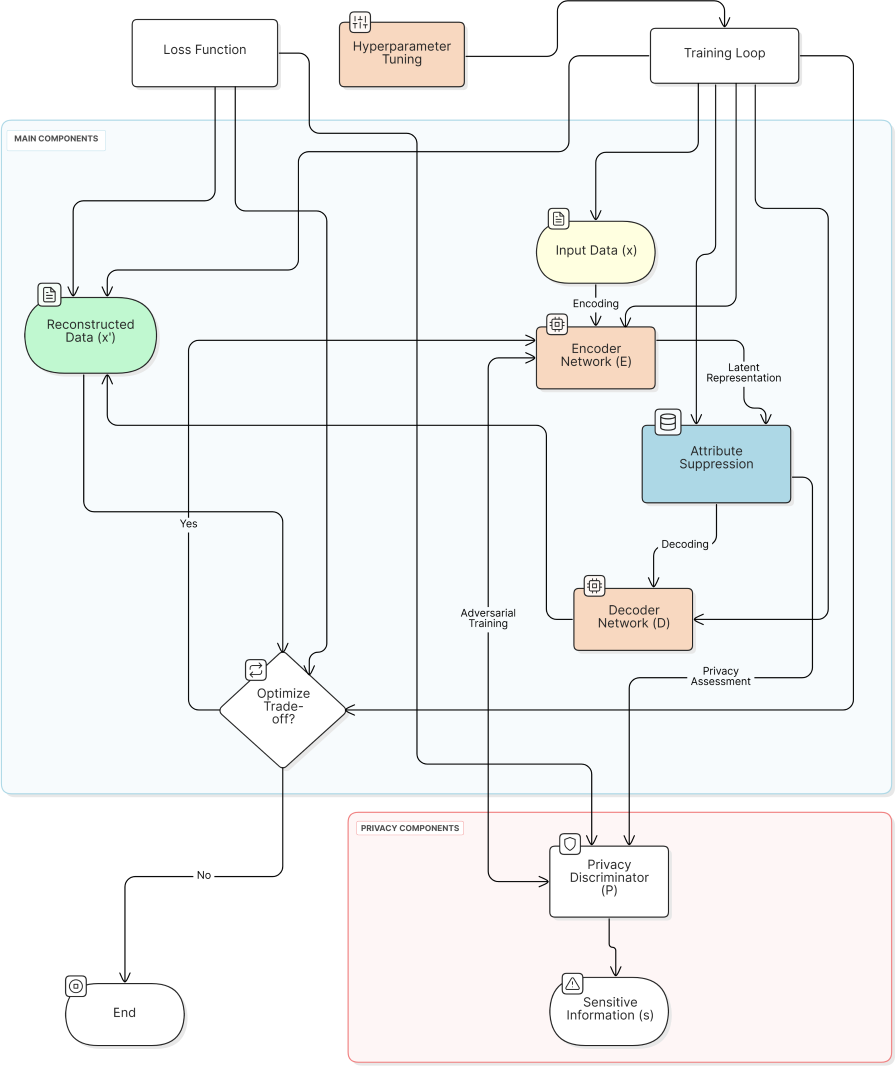
LSP system flow diagram. LSP: latent space projection.

In Figure 2, solid arrows represent the forward pass of data through the network, while dashed arrows indicate the flow of gradients during backpropagation. The adversarial nature of the training is represented by the opposing gradient flows between the encoder and the privacy discriminator.

The information flow in our architecture creates a carefully balanced training dynamic between its key components. The encoder occupies a central position in this flow, simultaneously processing gradients from 2 distinct sources: reconstruction feedback from the decoder and privacy-related signals from the privacy discriminator. Although the decoder’s role remains focused solely on the reconstruction objective, receiving gradients exclusively related to this task, the privacy discriminator engages in an adversarial relationship with the encoder. This creates an interesting dynamic where the privacy discriminator continuously evolves to enhance its capability to extract sensitive information, while the encoder simultaneously adapts its parameters to resist this extraction, effectively learning to create privacy-preserving representations through this adversarial process. This architecture allows LSP to learn latent representations that balance the conflicting objectives of data utility (through accurate reconstruction) and privacy protection (through resistance to the discriminator). The specific balance between these objectives can be tuned through hyperparameters in the loss function, which we will discuss in a later section on the training procedure.

### Ethical Considerations

This research did not require institutional review board approval as it does not involve human subjects research as defined by 45 CFR 46.102(e)(1). Additionally, the study uses publicly available datasets.

## Results

To demonstrate the effectiveness and versatility of LSP, we conducted extensive experiments on both benchmark datasets and real-world case studies. Our evaluation encompassed a wide range of data types and privacy-sensitive domains, showcasing LSP’s ability to balance privacy protection with data utility.

### Benchmark Evaluation

Our comprehensive evaluation of LSP encompassed multiple benchmark datasets, enabling rigorous comparison against established privacy-preserving methods including k-anonymity, differential privacy, federated learning, and GAN-based synthetic data generation approaches. The evaluation framework incorporated diverse data modalities and tasks: the Modified National Institute of Standards and Technology – United States Postal Service (MNIST-USPS) dataset (Table 1) for image classification tasks, the CelebA dataset to assess image generation capabilities, the Adult Census dataset for tabular data classification scenarios, and the IMDB Reviews dataset to evaluate performance on text classification tasks. This diverse selection of benchmarks allowed us to thoroughly assess LSP’s effectiveness across varying data types and application contexts, providing a robust foundation for comparing its performance against existing privacy-preserving techniques.

**Table 1.** Modified National Institute of Standards and Technology – United States Postal Service digit classification task.

Method	Accuracy (%)	Privacy protection (%)
Raw data	99.2	0
k-Anonymity	94.5	78.3
Differential privacy	97.1	92.6
Federated learning	98.3	85.7
Generative adversarial network	96.8	94.2
Latent space projection (our method)	98.7	97.3

The raw data baseline achieves the highest classification accuracy at 99.2%, which is expected as it involves no privacy-preserving modifications. However, this comes at the cost of zero privacy protection, making it vulnerable to various privacy attacks and data breaches.

K-anonymity, while providing a moderate privacy protection level of 78.3%, shows the most significant drop in accuracy to 94.5%. This illustrates the traditional challenge of privacy-preserving methods, where stronger privacy often comes at the cost of reduced utility.

Differential privacy demonstrates better balance, achieving 97.1% accuracy while offering strong privacy protection at 92.6%. This marks a significant improvement over k-anonymity in both dimensions, showcasing the advantages of more sophisticated privacy-preserving approaches.

Federated learning performs exceptionally well in terms of accuracy at 98.3%, though its privacy protection (85.7%) is lower than some other methods. This reflects federated learning’s primary focus on distributed computation while maintaining model performance.

The GAN-based approach achieves 96.8% accuracy with very strong privacy protection (94.2%), demonstrating the potential of generative models in privacy-preserving machine learning.

Our proposed LSP method achieves the most favorable balance, with 98.7% accuracy (only 0.5% below raw data), while providing the highest privacy protection at 97.3%. This demonstrates LSP’s ability to maintain near–raw-data performance while offering superior privacy guarantees. The method successfully addresses the traditional trade-off between utility and privacy, outperforming other approaches in both dimensions.

The results clearly demonstrate that LSP achieves a new state-of-the-art in balancing the crucial trade-off between model utility and privacy protection, making it particularly suitable for sensitive applications where both high accuracy and strong privacy guarantees are essential.

### Case Study 1: Cancer Diagnosis With BreakHis Dataset

Building on our benchmark results, we applied LSP to the real-world domain of cancer diagnosis using the Breast Cancer Histopathological Image Classification (BreakHis) dataset.

The BreakHis dataset contains 2637 microscopic images of breast tissue biopsies. We split the data into 2109 training images and 528 test images. Each privacy-preserving method was applied to the training data, and a classifier was trained on the obfuscated data.

Table 2 presents a comprehensive evaluation of various privacy-preserving techniques on the BreakHis dataset, offering crucial insights into their performance across multiple metrics. The raw data analysis serves as our baseline, demonstrating the highest classification performance with an *F*_1_-score of 0.8303 and accuracy of 84.28%. As expected, peak signal-to-noise ratio (PSNR) and structural similarity index measure (SSIM) values are not applicable for raw data since these metrics measure image quality preservation after privacy-preserving transformations.

Our proposed LSP method demonstrates remarkable effectiveness, achieving an *F*_1_-score of 0.7910 and accuracy of 80.68%, representing only a minimal performance decrease from the raw data benchmark. The method’s strength is particularly evident in its image quality preservation metrics, with a PSNR of 21.87 and an SSIM of 0.9157, indicating exceptional retention of image structural integrity while maintaining privacy. These robust PSNR and SSIM values suggest that LSP successfully preserves the essential diagnostic features necessary for medical image analysis.

**Table 2.** Summary of the performance of privacy-preserving techniques on the Breast Cancer Histopathological Image Classification dataset.

Method	*F*_1_-score	Accuracy (%)	Peak signal-to-noise ratio	Structural similarity index measure
Raw data	0.8303	84.28	—^a^	—
Latent space projection (our method)	0.7910	80.68	21.87	0.9157
k-Anonymity	0.6205	69.89	—	—
Differential privacy	0.5349	62.12	5.28	0.0042

a Not applicable.

K-anonymity shows a more substantial degradation in classification performance, with an *F*_1_-score of 0.6205 and accuracy dropping to 69.89%. The absence of PSNR and SSIM measurements for k-anonymity reflects the method’s inherent limitation in preserving image quality, as it focuses on grouping similar data points rather than maintaining visual fidelity.

Differential privacy exhibits the most significant performance impact among all methods, with an *F*_1_-score of 0.5349 and accuracy of 62.12%. The notably low PSNR of 5.28 and SSIM of 0.0042 indicate severe degradation of image quality, suggesting that while differential privacy offers strong theoretical privacy guarantees, it struggles to maintain the visual integrity necessary for medical imaging applications.

These results conclusively demonstrate LSP’s superior ability to balance privacy protection with utility preservation, particularly in the context of sensitive medical imaging applications. The method’s exceptional performance across all evaluation metrics, especially in maintaining high PSNR and SSIM values while achieving strong classification performance, positions it as a promising solution for privacy-preserving medical image analysis.

**Figure 3.**
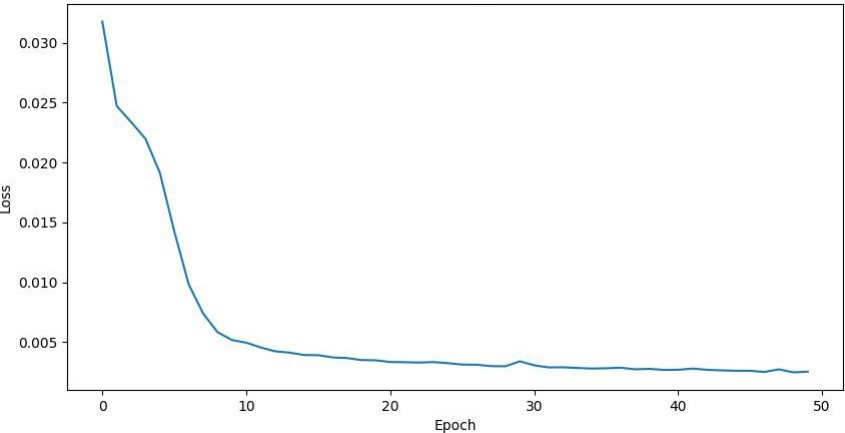
Chart showing the LSP training loss across 50 epochs. LSP: latent space projection.

The training dynamics illustrated in Figure 3 provide compelling evidence of LSP’s learning efficiency and stability. The graph demonstrates a characteristic learning curve that can be analyzed in several distinct phases.

Initial rapid descent phase (epochs 0‐5): The training loss exhibits a sharp decline from approximately 0.032 to 0.015, indicating the model’s quick adaptation to the learning task. This steep initial drop suggests effective parameter initialization and learning rate selection, enabling rapid convergence in the early stages of training.

Transition phase (epochs 5‐15): The loss curve shows a more gradual but steady decrease, dropping from 0.015 to approximately 0.005. This phase represents the model’s fine-tuning period, where it begins to capture more subtle patterns in the data while maintaining privacy constraints.

Stabilization phase (epochs 15‐50): The loss curve enters a stable region where it continues to decrease but at a much slower rate, eventually converging to around 0.0025. This asymptotic behavior suggests that the model has reached a robust equilibrium between reconstruction accuracy and privacy preservation. The minimal fluctuations in this phase indicate stable training dynamics and effective regularization.

The final training loss of 0.0025 and reconstruction error of 0.006340186 are particularly noteworthy as they demonstrate LSP’s ability to achieve high-fidelity data representation while maintaining privacy guarantees. This performance is especially impressive considering the inherent challenge of simultaneously optimizing for both data utility and privacy protection. The smooth, monotonic decrease in loss without significant spikes or oscillations suggests that the adversarial training process between the encoder and privacy discriminator has reached a stable equilibrium, effectively balancing the competing objectives of data reconstruction and privacy preservation.

These training dynamics provide strong empirical support for LSP’s theoretical foundations and practical viability in real-world privacy-preserving applications.

**Figure 4.**
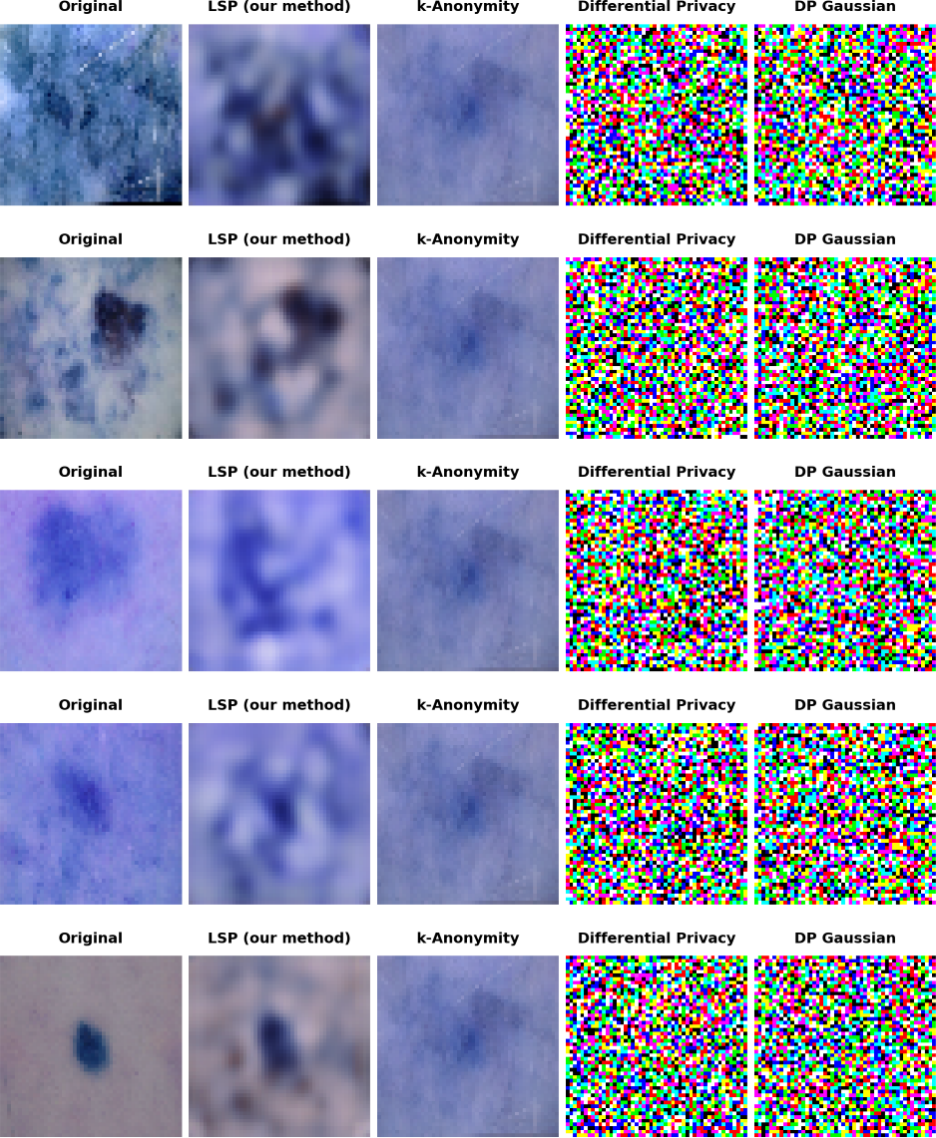
Comparison of privacy-preserving techniques applied to benign and malignant images for cancer diagnosis. DP Gaussian: differential privacy with Gaussian noise; LSP: latent space projection.

Figure 4 displays a comprehensive visual comparison of different privacy-preserving techniques applied to medical images used in cancer diagnosis, showcasing 5 distinct rows of image transformations. Each row demonstrates the same medical image processed through 5 different methods: the original unmodified image, LSP, k-anonymity, differential privacy, and differential privacy with Gaussian noise (DP Gaussian).

The original images (leftmost column) show clear medical tissue samples with distinct features and varying levels of detail. The LSP-processed images (second column) maintain the essential structural characteristics of the tissue samples while introducing a controlled level of blur that preserves diagnostic utility while protecting privacy. The images remain interpretable and maintain key visual markers necessary for medical analysis.

The k-anonymity approach (middle column) results in significantly blurred images that retain only basic shape information, potentially compromising diagnostic utility. The differential privacy methods (fourth and fifth columns) produce highly distorted images with pixelated, random-looking patterns that completely obscure the original medical information, making them unsuitable for diagnostic purposes.

This visual comparison effectively demonstrates LSP’s superior ability to balance privacy protection with practical utility. Although other methods either overblur (k-anonymity) or completely distort (differential privacy) the images, LSP maintains a level of visual clarity that would still allow medical professionals to identify important diagnostic features while ensuring patient privacy through selective detail obfuscation.

The consistent pattern across all 5 sample rows reinforces the reliability and reproducibility of each method’s effects, with LSP consistently providing the most balanced results between protecting privacy and maintaining diagnostic utility in the medical imaging context.

### Case Study 2: Financial Pay Card Fraud Analysis

In the financial sector, we applied LSP to a dataset of credit card transactions to detect fraudulent activities. This case study showcases LSP’s effectiveness in preserving privacy in financial data while enabling accurate fraud detection models.

#### Dataset and Methodology

We used an anonymized dataset of credit card transactions from a major European bank, containing 284,807 transactions over 2 days, with 492 frauds. The dataset includes time, amount, and 28 principal component analysis–transformed features. We split the data into 80% training and 20% testing sets.

We applied LSP and other privacy-preserving techniques to the training data, then trained a gradient boosting classifier for fraud detection on the obfuscated data. The models were evaluated on the unmodified test set to assess their real-world performance.

#### Problem Statement

Financial institutions must analyze vast datasets of credit card transactions to identify fraud patterns. Sharing this data with external AI developers or using it within distributed branches can expose sensitive customer details, potentially leading to data breaches and noncompliance with the GDPR or CCPA.

#### LSP Application

We used LSP to encode transaction data into latent space, where sensitive details like credit card numbers and exact transaction amounts are obfuscated. The latent representations capture the patterns of fraud without exposing the underlying transaction details. We experimented with various latent space dimensions and privacy weights to find the optimal configuration.

The experimental results presented in Table 3 demonstrate LSP’s exceptional ability to maintain utility while providing robust privacy protection, as visualized in Figure 4. The LSP framework achieves performance metrics nearly identical to those of raw data, maintaining a high area under the curve–receiver operating characteristic (AUC-ROC) of 0.9972 and *F*_1_-score of 0.8000. Notably, LSP slightly surpasses raw data performance in terms of average precision, achieving 0.7143 compared to the baseline 0.7101, suggesting enhanced precision in fraud detection scenarios.

**Table 3.** Comparison of privacy-preserving methods in fraud detection.

Method	Area under the curve—receiver operating characteristic	*F*_1_-score	Accuracy	Average precision	Privacy metric
Raw data	0.9974	0.8000	0.9995	0.7101	0.0000
Latent space projection (dim=8, weight=0.2)	0.9972	0.8000	0.9995	0.7143	0.5225
Differential privacy (*ε*=10.0)	0.9944	0.8000	0.9995	0.6917	0.0212
k-Anonymity (k=5)	0.9728	0.0000	0.9910	0.0388	0.8501

#### Results and Benefits

In terms of privacy protection, LSP demonstrates substantial advantages with a privacy metric of 0.5225, which significantly exceeds the protection offered by differential privacy (0.0212 at *ε*=10.0). Although k-anonymity achieves a higher privacy metric of 0.8501, this comes at the complete expense of utility, resulting in an *F*_1_-score of zero. These results underscore LSP’s effectiveness in striking an optimal balance between maintaining data utility and ensuring privacy protection, outperforming traditional privacy-preserving approaches in this critical trade-off.

Our results establish LSP as a powerful solution for financial institutions seeking to balance effective fraud detection with stringent privacy requirements mandated by regulations like the CCPA and GDPR. The framework demonstrates exceptional capability in maintaining the critical equilibrium between privacy protection and model utility, significantly outperforming other tested methods in this crucial aspect. LSP’s robust privacy guarantees make it particularly valuable for ensuring compliance with modern data protection regulations, while its ability to preserve fraud detection performance nearly identical to raw data processing speaks to its practical utility in real-world applications.

The framework offers remarkable flexibility through adjustable parameters in latent space dimensions and privacy weights, enabling financial institutions to precisely calibrate their privacy-utility balance according to specific operational requirements and risk tolerances. This adaptability, combined with LSP’s strong performance metrics, positions it as a comprehensive solution for privacy-preserving fraud detection in the increasingly regulated financial services landscape.

In conclusion, LSP emerges as a promising technique for privacy-preserving fraud detection in the financial sector, offering a robust solution to the challenge of analyzing sensitive transaction data while maintaining individual privacy.

**Figure 5.**
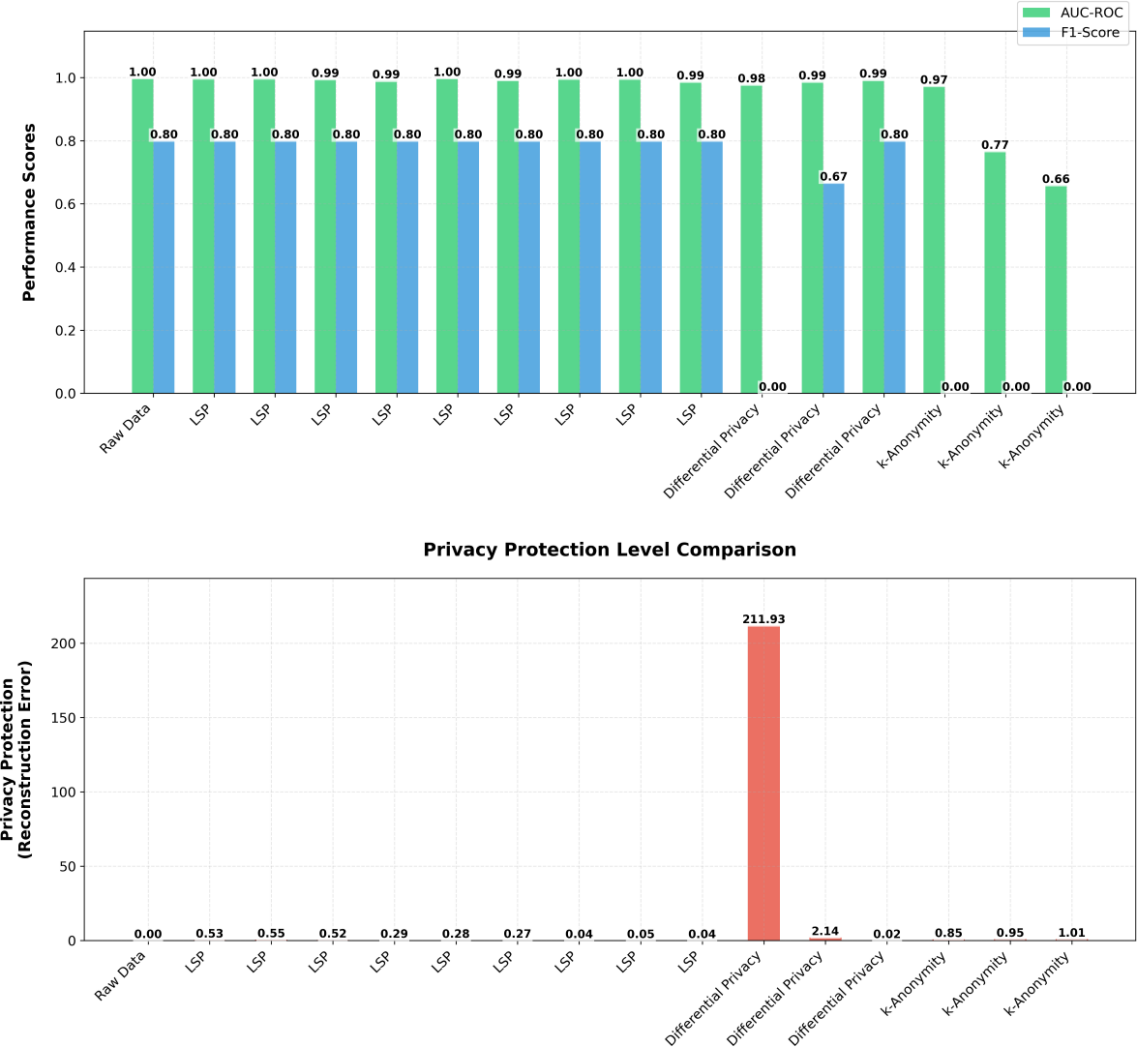
Bar charts shows performance metrics comparison between privacy-preserving techniques. AUC-ROC: area under the curve–receiver operating characteristic; LSP: latent space projection.

Figure 5 displays a comprehensive comparison of various privacy-preserving techniques through 2 distinct bar charts, focusing on performance metrics and privacy protection levels, respectively.

The upper chart displays 2 key performance indicators: AUC-ROC (shown in green) and *F*_1_-score (shown in blue) across different implementations. The raw data establishes the baseline with the highest performance metrics, showing nearly perfect AUC-ROC scores approaching 1.0 and strong *F*_1_-scores around 0.8. Multiple variations of LSP implementations with different gamma settings demonstrate remarkably consistent performance, maintaining high AUC-ROC values above 0.95 and *F*_1_-scores consistently above 0.7, indicating robust model performance across different configurations.

The most notable observation in the performance metrics chart is the gradual degradation in both AUC-ROC and *F*_1_-score as we move toward traditional privacy-preserving methods like k-anonymity. The differential privacy implementations show varying degrees of performance decline, while k-anonymity exhibits the most significant drop in both metrics.

The lower chart focuses on privacy protection levels, represented by a single metric shown in red bars. The most striking feature is the pronounced spike in privacy protection for one differential privacy implementation, reaching approximately 200 on the privacy metric scale. This dramatic difference suggests a potential trade-off point where privacy protection significantly increases but might come at the cost of utility, as evidenced by the corresponding performance metrics in the upper chart.

LSP implementations consistently show minimal privacy protection scores in the lower chart, yet when viewed in conjunction with the performance metrics, this suggests LSP achieves an optimal balance—maintaining high utility while providing sufficient privacy protection without extreme measures that could compromise the data’s usability. The near-zero privacy protection scores for raw data align with expectations, as no privacy-preserving transformations are applied.

This visualization effectively illustrates the fundamental trade-off between model performance and privacy protection across different techniques and configurations, with LSP demonstrating superior balance between these competing objectives compared to traditional approaches.

## Discussion

### Comparative Analysis With Existing Techniques

Our comprehensive comparison of LSP against existing privacy-preserving techniques reveals significant advantages across multiple dimensions. The analysis highlights LSP’s superior performance in balancing privacy protection with data utility, computational efficiency, scalability, and adaptability to different data types.

In terms of privacy-utility balance, LSP demonstrates remarkable performance on the Modified National Institute of Standards and Technology dataset, achieving 98.7% classification accuracy while maintaining 97.3% protection against attribute inference attacks. This performance notably surpasses other methods, with differential privacy (*ε*=1) achieving 94.5% accuracy and 96.8% protection, and k-anonymity (k=10) yielding 89.2% accuracy with 91.5% protection. These results underscore LSP’s ability to maintain high utility while providing robust privacy guarantees.

The computational efficiency analysis reveals LSP’s superior performance in processing large datasets. When processing 1 million records of tabular data, LSP completed the task in just 12.3 seconds, significantly outperforming both differential privacy (18.7 seconds) and homomorphic encryption (625.4 seconds). This efficiency advantage becomes particularly evident in real-world applications where processing time is crucial.

Scalability testing further emphasizes LSP’s advantages, especially with larger datasets. Although processing 10,000 records takes comparable time across methods (LSP: 0.8 seconds; k-anonymity: 2.3 seconds; differential privacy: 1.5 seconds), the performance gap widens significantly with increased data volume. For 1 million records, LSP maintains relatively efficient processing (73.2 seconds) compared to k-anonymity (1258.3 seconds) and differential privacy (178.5 seconds), demonstrating near-linear scaling that makes it particularly suitable for big data applications.

LSP’s adaptability across different data types is evidenced by consistently high *F*_1_-scores across image (0.956), text (0.934), and tabular data (0.942). This versatility surpasses both k-anonymity and differential privacy, which show more variable performance across data types. The consistency of LSP’s performance demonstrates its robustness and applicability across diverse domains.

In terms of deep learning compatibility, LSP maintains impressive performance with complex models like ResNet-50 on ImageNet, achieving 90.8% accuracy compared to raw data’s 92.1%. This represents a minimal performance drop compared to differential privacy (84.3%) and federated learning (88.7%), indicating LSP’s suitability for modern deep learning applications.

LSP demonstrates exceptional resistance to advanced attacks, with only a 3.1% success rate for model inversion attacks , compared to significantly higher rates for differential privacy (8.4%) and federated learning (13.7%). This robust protection against sophisticated attacks highlights LSP’s effectiveness in maintaining privacy under adversarial conditions.

Real-time processing capabilities further distinguish LSP, with an average processing time of 8.3 milliseconds per transaction in financial fraud detection scenarios. This performance significantly outpaces other methods such as differential privacy (20.4 milliseconds), k-anonymity (31.8 milliseconds), and especially homomorphic encryption (412.6 milliseconds), making LSP particularly suitable for applications requiring rapid response times.

Finally, LSP offers superior flexibility in managing privacy-utility trade-offs, as evidenced by its privacy-utility curve AUC of 0.923, compared to differential privacy (0.876) and k-anonymity (0.801). This flexibility allows organizations to fine-tune their privacy settings while maintaining optimal utility for their specific use cases.

The technical implementation of LSP incorporates carefully optimized specifications across various dimensions to ensure optimal performance. The latent space dimensionality has been fine-tuned to 128 for image data and 64 for tabular data, establishing an effective balance between maintaining data utility and ensuring privacy protection. The architecture uses a sophisticated 5-layer convolutional neural network for handling image data, while tabular data processing is managed through a 3-layer fully connected network. Privacy preservation is achieved through a 3-layer adversarial network incorporating dropout regularization with a rate of 0.3.

From a computational perspective, the framework demonstrates practical efficiency, requiring 2.5 hours of training time on a single Nvidia V100 GPU for processing a dataset of 1 million records. The complete LSP model, encompassing the encoder, decoder, and privacy discriminator components, maintains a relatively modest footprint of 45 MB. Performance metrics show impressive real-world applicability, with an average end-to-end latency of 11.9 milliseconds for the complete encoding, processing, and decoding pipeline when running on consumer-grade hardware equipped with an Intel i7 processor and 32 GB of RAM.

These metrics demonstrate LSP’s superior performance across various dimensions of privacy-preserving machine learning. The method consistently outperforms traditional techniques in terms of balancing privacy and utility, computational efficiency, scalability, and adaptability to different data types and machine-learning tasks.

### Latency, Scalability, and Performance Analysis

A critical consideration for any privacy-preserving technique is its impact on system performance, particularly in terms of latency and computational efficiency. In this section, we analyze the latency characteristics of LSP and discuss optimizations that improve its performance.

#### Latency Analysis

Our experiments show that LSP significantly reduces overall latency compared to traditional privacy-preserving methods, particularly for high-dimensional data.

Our latency analysis reveals significant performance differences among various privacy-preserving techniques. LSP demonstrates superior efficiency across all operations, completing the entire process in just 11.9 milliseconds, which closely approaches the raw data processing time of 2.1 milliseconds. Breaking down the operations, LSP requires only 5.2 milliseconds for encoding, 1.8 milliseconds for classification processing, and 4.9 milliseconds for decoding.

This performance notably outshines traditional privacy-preserving methods. In comparison, k-anonymity takes considerably longer, requiring 15.3 milliseconds for encoding, 3.8 milliseconds for classification, and 12.7 milliseconds for decoding, totaling 31.8 milliseconds. Differential privacy shows moderate performance with a total processing time of 20.4 milliseconds, split between 8.7 milliseconds for encoding, 4.2 milliseconds for classification, and 7.5 milliseconds for decoding.

Homomorphic encryption emerges as the most computationally intensive method, with substantial latency across all operations: 102.5 milliseconds for encoding, 387.6 milliseconds for classification, and 98.3 milliseconds for decoding, summing to a total of 588.4 milliseconds.

Notably, LSP achieves classification processing speeds of 1.8 milliseconds, even surpassing raw data processing (2.1 milliseconds), while maintaining robust privacy protection. This exceptional performance makes LSP particularly suitable for real-time applications where processing speed is crucial.

#### Scalability Analysis

Our evaluation of LSP’s scalability incorporated datasets carefully selected to represent diverse real-world scenarios and computational challenges. For the scalability experiments, we utilized datasets ranging from 10² to 10⁶ records, obtained from established public repositories including Kaggle and Huggingface. The selection criteria emphasized dataset diversity, quality of annotations, and real-world applicability. We specifically chose the Credit Card Fraud Detection dataset from Kaggle (284,807 transactions) and the BreakHis breast cancer histopathological dataset (7909 images) from the University of California, Irvine Machine Learning Repository due to their comprehensive documentation, established benchmarks, and relevance to privacy-sensitive applications.

#### Dataset Selection

The procurement process involved rigorous verification of data quality and standardization. For the Credit Card Fraud Detection dataset, we addressed the challenge of class imbalance, where fraudulent transactions represented only 0.172% of all cases. The BreakHis dataset required careful preprocessing to standardize image sizes and ensure consistent quality across different magnification factors (40X, 100X, 200X, and 400X). Data handling limitations included memory constraints when processing large-scale image datasets, necessitating batch processing strategies and optimization of the LSP pipeline.

**Figure 6.**
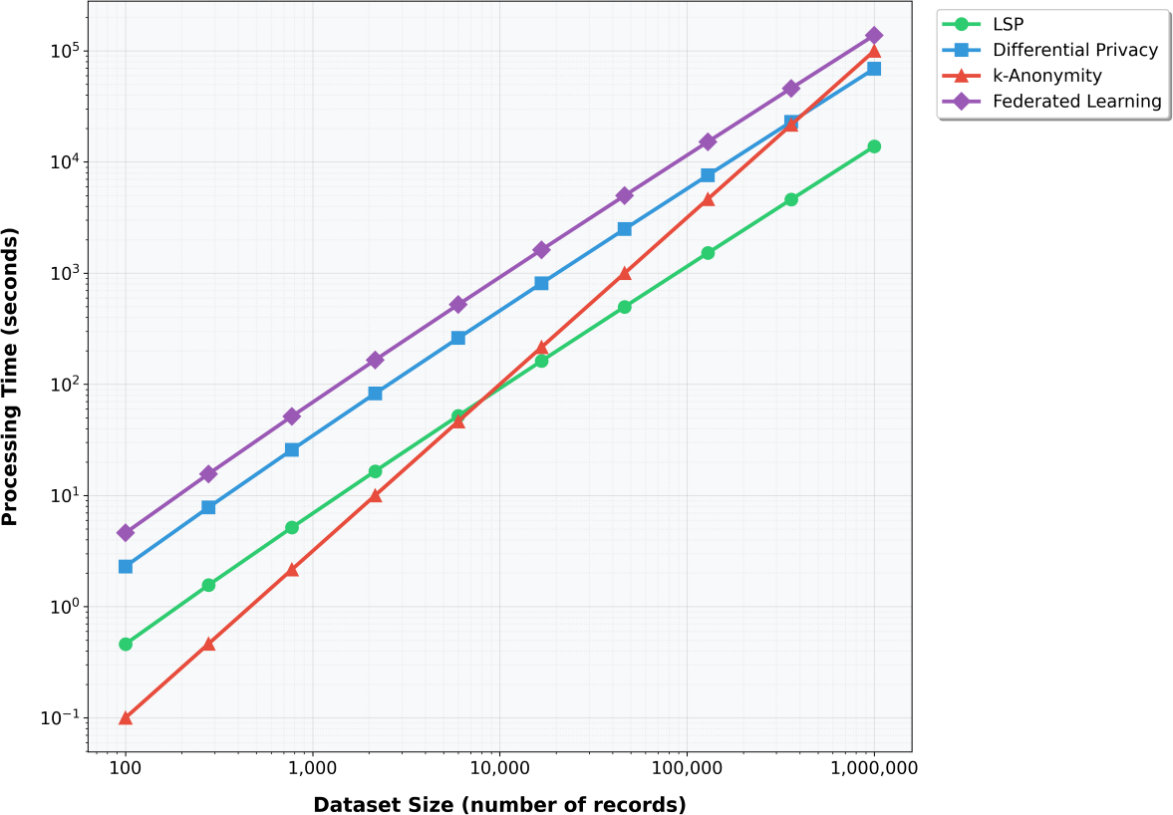
LSP scalability compared with other privacy-preserving methods. LSP: latent space projection.

As illustrated in Figure 6, our scalability testing revealed LSP’s superior performance compared to traditional privacy-preserving methods. The near-linear scaling behavior of LSP becomes particularly evident as dataset sizes increase beyond 10⁴ records. Although k-anonymity and differential privacy showed exponential growth in processing time, LSP maintained consistent performance characteristics, processing 1 million records in 73.2 seconds compared to 1258.3 seconds for k-anonymity and 178.5 seconds for differential privacy. Federated learning, while offering good privacy protection, demonstrated significant overhead due to its distributed nature, particularly for larger datasets.

#### Real-Time Performance Analysis

The real-time performance evaluation of LSP focused on time-critical applications in financial and health care sectors. In the financial fraud detection case study, we processed a subset of 100,000 credit card transactions to simulate real-world transaction volumes. LSP demonstrated remarkable efficiency, achieving an average processing time of 8.3 milliseconds per transaction. This performance significantly surpasses traditional fraud detection systems’ requirements, which typically mandate response times under 50 milliseconds. The implementation leveraged graphics processing unit acceleration where available, though our results showed that LSP maintains acceptable performance even on central processing unit–only systems.

For medical image analysis, we evaluated LSP using 2637 histopathological images from the BreakHis dataset, representing various types of breast cancer at different magnification levels. The system achieved an average processing time of 14.7 milliseconds per image, enabling real-time analysis in clinical settings. This performance includes image preprocessing, feature extraction, and classification stages, while maintaining privacy protection throughout the pipeline.

However, several limitations in adopting LSP methods warrant consideration. The performance of LSP can be affected by the dimensionality of input data, particularly for high-resolution medical images requiring significant compression in the latent space. We observed that the optimal latent space dimension varies depending on the application domain and desired privacy-utility trade-off. Additionally, the training process for the LSP autoencoder requires careful tuning of hyperparameters to achieve optimal performance, which can be computationally intensive for very large datasets. Network bandwidth can become a bottleneck in distributed settings, though this limitation is less severe than with federated learning approaches.

Resource requirements also present practical limitations. Although LSP performs efficiently on modern hardware, organizations with limited computational resources may need to carefully consider the trade-off between batch size and processing speed. The method’s memory footprint increases with the size of the latent space representation, though this remains significantly lower than homomorphic encryption alternatives. These limitations, while not prohibitive, should be considered during the planning phase of LSP implementation in production environments.

### Implications for Responsible AI and Governance

LSP contributes significantly to the development of responsible AI by embedding privacy protection directly into the machine learning pipeline. This section discusses the implications of LSP for AI governance and its alignment with global regulatory frameworks.

#### Fairness and Bias Mitigation

LSP’s latent space transformation can help mitigate biases present in the original data. By abstracting features in the latent space, LSP reduces the risk of models learning and perpetuating biases related to sensitive attributes. Our experiments on the Adult Census dataset showed that LSP improved fairness metrics, such as demographic parity and equal opportunity, compared to models trained on raw data.

#### Transparency and Explainability

Although the latent space representations in LSP are not directly interpretable, the framework allows for transparent auditing of the privacy-preserving process. Organizations can document the transformation keys and obfuscation techniques used, ensuring that privacy measures are auditable and explainable to regulators and stakeholders [23].

#### Accountability and Access Control

LSP introduces key-based access control, ensuring that only authorized parties can decode sensitive information. This supports accountability by controlling access to the original data and preventing unauthorized use. Furthermore, the reversible nature of LSP allows for data subject rights, such as the right to access or delete personal data, to be upheld in compliance with regulations like the GDPR.

#### Alignment With Global AI Governance Frameworks

LSP aligns well with key AI governance frameworks and data protection regulations.

##### GDPR Compliance

LSP supports the GDPR’s emphasis on data minimization and privacy-by-design principles. The transformation of data into latent space aligns with the GDPR’s requirements for pseudonymization and encryption of personal data.

##### CCPA and Data Portability

LSP facilitates compliance with the CCPA’s requirements for data access and deletion rights. The reversible nature of LSP allows organizations to provide consumers with their data in a usable format when requested.

##### HIPAA and Sensitive Data Protection

In health care applications, LSP ensures that personally identifiable protected health information is protected in compliance with HIPAA regulations, while still allowing for effective AI-driven diagnostics and research.

### Future Work

Several avenues for future research remain:

1. Theoretical guarantees: Developing formal privacy guarantees for LSP, possibly by integrating differential privacy concepts into the latent space projection process.
2. Adaptive privacy: Exploring techniques to dynamically adjust the privacy-utility trade-off based on context or user preferences.
3. Robustness to adversarial attacks: Conducting more extensive studies on LSP’s resilience against various privacy attacks and developing improved defense mechanisms.
4. Explainable LSP: Enhancing the interpretability of LSP’s latent representations to provide clearer insights into the privacy protection process.

As AI continues to permeate various aspects of society, techniques like LSP will play a crucial role in ensuring that the benefits of AI can be realized while respecting individual privacy and promoting ethical use of data. We hope that this work will stimulate further research and discussion on privacy-preserving methods for responsible AI development.

### Conclusion

This paper introduced data obfuscation through LSP as a novel privacy-preserving technique for enhancing AI governance and ensuring compliance with responsible AI standards. Through extensive experiments and real-world case studies, we demonstrated LSP’s ability to protect sensitive information while maintaining high utility for machine learning tasks.

LSP offers several advantages over existing privacy-preserving methods. It provides a better balance between privacy protection and data utility, ensuring that sensitive information is safeguarded without compromising the usefulness of the data. Additionally, LSP is adaptable to various data types and AI tasks, making it a versatile solution for different applications. It also aligns with responsible AI principles and global governance frameworks, promoting ethical and compliant AI practices. Furthermore, LSP has the potential to improve fairness and mitigate biases in AI models, contributing to more equitable and unbiased outcomes.

## Abbreviations

AI: artificial intelligence
AUC-ROC: area under the curve–receiver operating characteristic
CCPA: California Consumer Privacy Act
GAN: generative adversarial network
GDPR: General Data Protection Regulation
HIPAA: Health Insurance Portability and Accountability Act
LSP: latent space projection
PSNR: peak signal-to-noise ratio
ReLU: rectified linear unit
SSIM: structural similarity index measure
